# Dicer Is Associated with Ribosomal DNA Chromatin in Mammalian Cells

**DOI:** 10.1371/journal.pone.0012175

**Published:** 2010-08-13

**Authors:** Lasse Sinkkonen, Tabea Hugenschmidt, Witold Filipowicz, Petr Svoboda

**Affiliations:** Friedrich Miescher Institute for Biomedical Research, Basel, Switzerland; Texas A&M University, United States of America

## Abstract

**Background:**

RNA silencing is a common term for pathways utilizing small RNAs as sequence-specific guides to repress gene expression. Components of the RNA silencing machinery are involved in different aspects of chromatin function in numerous organisms. However, association of RNA silencing with chromatin in mammalian cells remains unclear.

**Methodology/Principal Findings:**

Immunostaining of mitotic chromosomes with antibodies visualizing either endogenous or ectopically expressed Dicer in mammalian cells revealed association of the protein with ribosomal DNA (rDNA) repeats. Chromatin immunoprecipitations and bisulfite sequencing experiments indicated that Dicer is associated with transcribed regions of both active and silenced genes in rDNA arrays of interphase chromosomes. Metabolic labeling of the mouse embryonic stem (ES) cells lacking Dicer did not reveal apparent defect in rRNA biogenesis though pre-rRNA synthesis in these cells was decreased, likely as a consequence of their slower growth caused by the loss of miRNAs. We analyzed in detail chromatin structure of rDNA but did not find any epigenetic changes at rDNA loci in Dicer^−/−^ ES cells. Instead, we found that rDNA methylation is rather low in primary tissues, contrasting with rDNA methylation patterns in transformed cell lines.

**Conclusion/Significance:**

We found that Dicer, a key component of RNA silencing pathways, can be detected in association with rDNA chromatin in mammalian cells. The role of this particular localization of Dicer is not readily apparent since the enzyme is associated with rDNA genes regardless of their transcriptional activity. However, localization of Dicer to the transcribed region suggests that transcription may contribute to the Dicer deposition at rDNA chromatin. We hypothesize that Dicer functions in maintaining integrity of rDNA arrays.

## Introduction

RNA interference (RNAi) and microRNA (miRNA) pathways represent RNA silencing mechanisms utilizing short RNA molecules, produced by RNAse III family enzyme Dicer, to guide sequence-specific silencing of gene expression. Factors involved in RNA silencing also participate in the formation and maintenance of heterochromatin. The connection between RNA silencing and chromatin is best established for *Schizosaccharomyces pombe* and plants where chromatin-associated silencing complexes were extensively characterized (reviewed in [1], [2]). Components of RNA silencing were implicated in transcriptional silencing and heterochromatin also in animals but, unlike in plants and *S. pombe*, the mechanistic aspects of the connection remain unclear [3]. In mammals, transfected siRNAs were reported to cause heterochromatinization of targeted genes in a process possibly involving Argonaute proteins [4]–[6]. In addition, mammalian Dicer was implicated in the formation of centromeric heterochromatin [7], [8], and in regulation of intergenic transcription at the human and chicken β-globin locus [9], [10]. However, recent data indicate that some of the Dicer effects on epigenetic mechanisms can be indirectly mediated by miRNAs, what warrants caution when interpreting results obtained with cells depleted of Dicer or Argonaute proteins [11], [12].

The uncertainty about small-RNA mediated epigenetic silencing in mammals contrasts with experimentally supported model of heterochromatin formation in *S. pombe* where Dicer cleaves transcripts from centromeric and other repeats to produce short RNAs that recruit complexes inducing local formation of heterochromatin (reviewed in [2]). Other RNA silencing factors such as Ago1 and Rdp1 operate in *cis* as stable components of heterochromatin at centromeres, telomeres, mating type locus, and ribosomal DNA (rDNA) repeats [13]. Since mutations of RNAi machinery components lead to increased mitotic recombination of rDNA repeats in *S. pombe*, the RNAi pathway may also control integrity of rRNA gene arrays [13]. A similar role has been proposed also for quelling, an RNA silencing mechanism in *Neurospora crassa* [14].

Mammalian ribosomal RNA (rRNA) genes are organized, like in other organisms, in tandemly-repeated arrays. Human rDNA comprise approximately four hundred 43-kb repeats composed of a 13-kb transcribed region and a 30-kb intergenic spacer (IGS). Human rDNA arrays are localized together with satellite repeats on short arms of acrocentric chromosomes [15]. Mouse rDNA has a similar organization [16]. Transcriptional and epigenetic regulation of rDNA has been extensively studied (reviewed in [16], [17]). In established mammalian cell lines, approximately half of rDNA promoters are inactive and methylated [16], [18]. Silencing of rDNA is mediated by the chromatin nucleolar remodeling complex (NoRC), which is guided by 150–300 nucleotide-long noncoding RNAs (pRNAs) that are complementary to the rDNA promoter. Mutations that abrogate RNA binding of TIP5, the large subunit of NoRC, impair association of NoRC with rDNA and fail to promote histone H3 lysine 9 (H3K9) and and histone H4 lysine 20 (H4K20) methylation and HP1 protein recruitment. Knockdown of pRNAs abolishes the nucleolar localization of NoRC, decreases DNA methylation, and enhances rDNA transcription [19], [20]. Connection between pRNAs and RNA silencing pathways is uncertain as analysis of rDNA-derived small RNAs provided no evidence that pRNAs are processed by Dicer [21].

However, several links between RNA silencing and rRNA expression in animals can be found in the literature. Drosha, a mammalian RNase III family enzyme responsible for processing of primary miRNA transcripts to precursor miRNAs, was implicated in pre-rRNA processing [22] though reports contradicting this possibility have also appeared [23]. rRNA fragments are always found among cloned small 20-25-nt-long RNAs although they do not seem to stem from Dicer processing and their physiological role remains unclear [21], [24]. Interestingly, Peng and Karpen have reported decreased H3K9 dimethylation and increased recombination of repetitive DNA, including rDNA, in Drosophila mutants in Su(var)3-9 and Dicer-2 genes, suggesting that RNA silencing pathway is also involved in modulating rDNA chromatin in insects [25].

Here, we report that Dicer is physically associated with rDNA repeats on chromosomes of human and mouse cells. Analysis of rDNA chromatin and rRNA biogenesis suggests that Dicer is not directly regulating expression or processing of rRNA in mammalian cells. Possibly, Dicer association with rDNA chromatin is indicative of its role in maintenance of rDNA tandem repeats. Such a role would be consistent with the fact that Dicer associates with both active and silenced rDNA genes throughout the cell cycle. In addition, our data provide an insight into epigenetic status of rRNA genes in mouse embryonic stem (ES) cells and different tissues.

## Results

### Localization of Dicer on mammalian chromosomes

**Figure 1:**
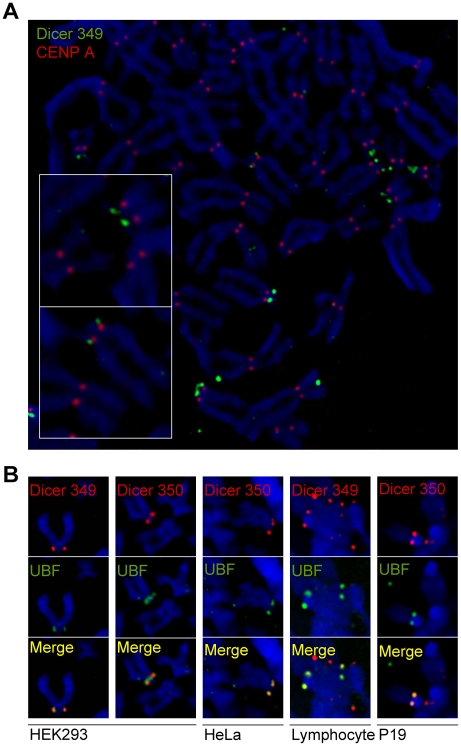
Localization of Dicer on mammalian mitotic chromosomes. Chromosome spreads from human or mouse cells were stained with anti-Dicer and other indicated antibodies. (A) Staining of HEK293 chromosomes with anti-CENP-A (red) and anti-Dicer D349 (green) antibodies. The p-arms of acrocentric chromosomes stained by D349 antibody are indicated by arrows. The inset shows an example of a distinctly shaped acrocentric chromosome with a pair of dots Dicer staining on p-arms. (B) Pairs of dots stained with anti-Dicer antibodies D349 (left column) and D350 (right column) (red) co-localize with dots stained by anti-UBF antibody (green). The UBF staining co-localized with the D349 and D350 antibody stainings in 95,4% (n = 65) and 93,5% (n = 62) of all inspected HEK293 chromosomes, respectively. Likewise, pairs of dots stained with anti-Dicer antibodies D349 or D350 (red) co-localize with dots stained by anti-UBF antibody (green) on mitotic chromosomes prepared from HeLa cells, human primary lymphocytes, and mouse teratocarcinoma P19 cells.

We have immunostained mitotic chromosomes of human HEK293 cells using two polyclonal antibodies, D349 and D350, raised against different epitopes of human Dicer (Fig. S1). Both antibodies produced characteristic “pair of dots” signals at short (p) arms of a set of distinctly shaped chromosomes, recognized as acrocentric chromosomes 13, 14, 15, 21, and 22 (Fig. 1). Co-staining for CENP-A, a centromere-specific protein, demonstrated that signals visualized with anti-Dicer antibodies were close to but not overlapping with the pairs of CENP-A-specific signals, consistent with the Dicer localization on p-arms of acrocentric chromosomes (Fig. 1A).

To verify that Dicer is localized at rDNA loci, we co-stained mitotic chromosomes with antibodies against UBF, an RNA polymerase I factor involved in rRNA transcription [16]. Signals detected with both anti-Dicer antibodies co-localized with those specific for UBF (Fig. 1B). A similar pattern of Dicer and UBF co-localization was also observed with mitotic chromosomes prepared from human lymphocytes and HeLa cells, and mouse P19 cells (Fig. 1B).

**Figure 2:**
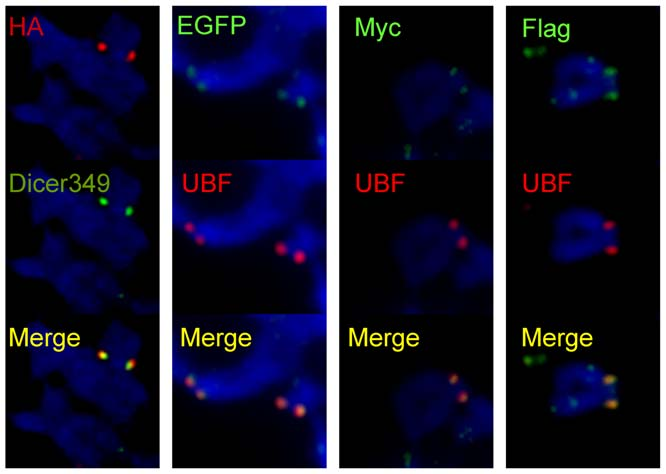
Localization of tagged Dicer proteins on mammalian mitotic chromosomes. Transiently expressed tagged Dicer proteins are detected at rDNA loci. Constructs expressing proteins bearing different tags at the N terminus were transfected to HEK293 cells and the tag was visualized by anti-EGFP, anti-HA, anti-Flag, or anti-Myc antibodies.

Specificity of rDNA staining with anti-Dicer antibodies was confirmed using expression plasmids expressing HA-, EGFP-, Flag-, or Myc-tagged Dicer. Transiently expressed tagged Dicer detected by anti-EGFP, anti-HA, anti-Flag, or anti-Myc antibodies also colocalized with UBF signal (Fig. 2), further confirming that Dicer is physically present on mitotic rDNA chromatin in mammalian cells.

### Specific association of Dicer with transcribed and promoter regions of rDNA repeats

**Figure 3:**
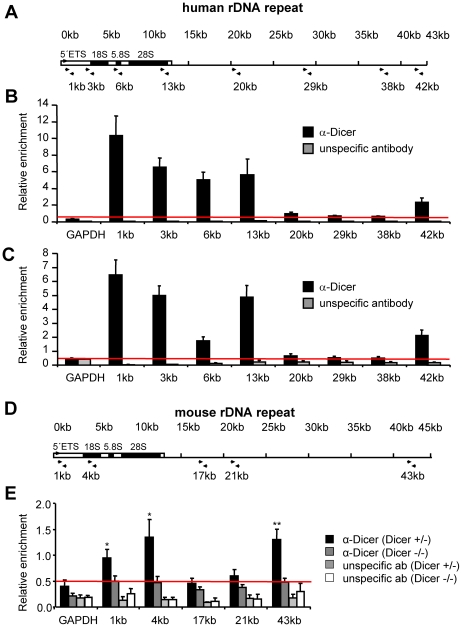
Association of Dicer with transcribed and promoter regions of mammalian rDNA repeats. (A) Structure of the human rDNA repeat and positions of primers used for the ChIP analysis. (B) ChIP analysis performed with HEK293 cells using Dicer D349 antibody and an unspecific antibody as a control. Real-time quantitative PCR was performed with the immunoprecipitated DNA using primer pairs indicated in panel A. The glyceraldehyde-3-phosphate dehydrogenase (GAPDH) primers were located in the gene promoter region. Values are calculated as percentage of the input DNA used at 1∶100 dilution. They represent means (+/− SEM) of at least 4 independent experiments. The red line represents the threshold of the 0.5% of input level, above which enrichments were considered as significant. (C) ChIP analysis performed with G1-phase HEK293 cells shows pattern of enrichment of Dicer on rDNA repeats similar to that seen with total population of HEK293 cells. Values represent means (+/− SEM) of at least 3 independent experiments. For other details, see panel B. (D) Structure of the mouse rDNA repeat and positions of primers used for the ChIP analysis. (E) Loss of rDNA enrichment by the Dicer D349 antibody in mouse Dicer knockout ES cells. The ChIP was performed with mouse Dicer^+/−^ and Dicer^−/−^ ES cells similarly as with HEK293 cells. Real-time quantitative PCR was performed with the precipitated DNA using primer pairs indicated in panel D. Values represent means (+/− SEM) of at least 7 independent experiments. Statistical significance of the drop in enrichment at 1-kb, 4-kb and 43-kb regions was determined by two-tailed t-test and the p-values were 0.015, 0.026 and 0.003, respectively. For other details, see panel B.

To delineate Dicer localization in rDNA more precisely, we performed chromatin immunoprecipitation (ChIP) analysis in HEK293 cells with the anti-Dicer D349 antibody and used the immunoprecipitated DNA for real-time PCR analysis with primer pairs covering different regions of the human rDNA repeat (Fig. 3A). We found a specific enrichment at the promoter (42-kb region) and throughout the transcribed part of rDNA (1-, 3-, 6-, and 13-kb regions), with the highest peak at around 1-kb downstream of the transcription start site. In IGS, the enrichment was very low (Fig. 3B). Similar results were obtained using HEK293 cells in the G1-phase of cell cycle, indicating that the association of Dicer is not specific to M-phase when nuclear and cytoplasmic compartments are not separated by nuclear membrane (Fig. 3C). ChIP experiments performed with HeLa cells also showed Dicer enrichment at rDNA promoter and transcribed region (data not shown). Analysis with primers specific for β-satellite and satellite III sequences that surround the rDNA arrays [15] indicated no Dicer-specific enrichment exceeding that seen at the rDNA intergenic spacer (data not shown).

To obtain additional support for the association of Dicer with mammalian rDNA chromatin, we have analyzed Dicer-deficient (Dicer^−/−^) mouse ES cells, using heterozygous (Dicer^−+/−^) ES cells as a control [26]. ChIP analysis performed with anti-Dicer D349 antibody revealed enrichment of rDNA in Dicer expressing mouse ES cells although enrichment was lower than in human cells (Fig. 3D, E). However, similarly to human cells, the antibody preferentially enriched promoter and transcribed regions (1-, 4-, and 43-kb), but no intergenic 17-kb and 21-kb mouse rDNA regions (Fig. 3D, E). Most importantly, the ChIP analysis performed with Dicer^−/−^ cells showed a statistically significant drop to background level in the enrichment of 1-, 4-, and 43-kb regions (Fig. 3E), indicating that the D349 antibody monitors a conserved association of Dicer with rDNA. Analysis of chromosome spreads from mouse ES cells by indirect immunofluorescence did not yield reliable staining of rDNA with Dicer D349 and D350 antibodies as the signal was very weak and difficult to distinguish from background staining (data not shown). This is consistent with a lower enrichment of rDNA in ChIP experiments performed with mouse ES cells and could possibly be due to a low level of Dicer associated with rDNA in ES cells or a lower Dicer expression in these cells when compared to HEK293 cells (Fig. S1B and C).

### Dicer associates with active and inactive rRNA genes

**Figure 4:**
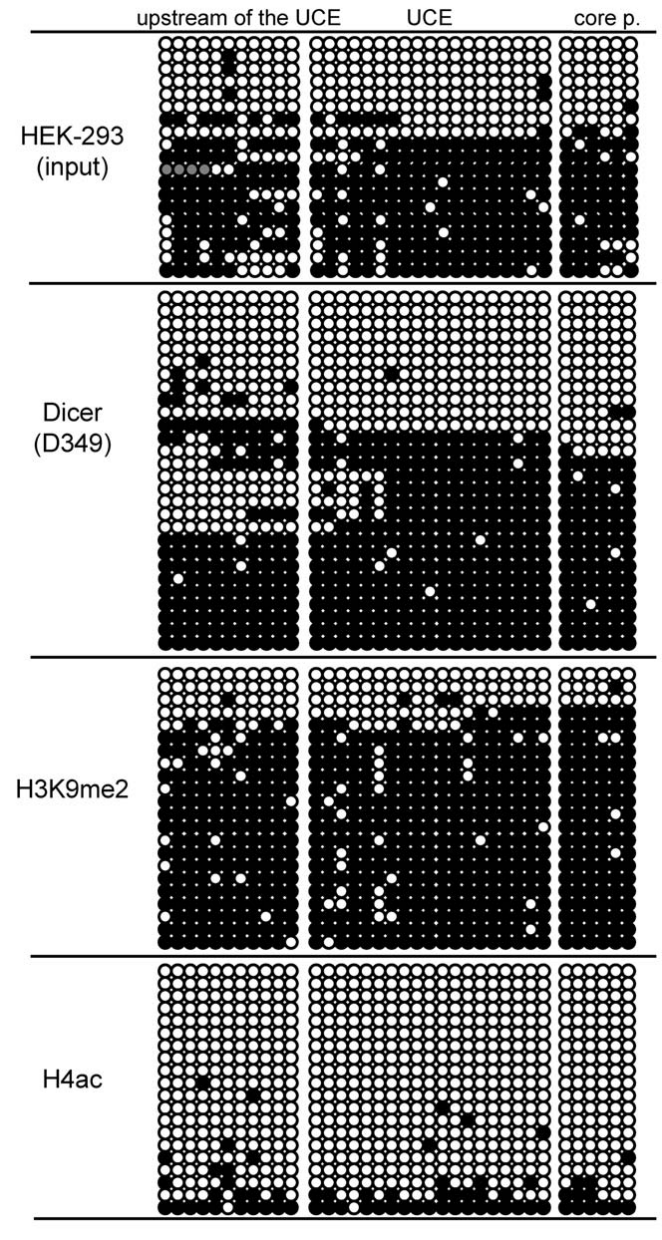
Methylation status of the Dicer associated rDNA as revealed by bisulfite sequencing. The sequenced region, spanning positions –186 to +20 of the rDNA repeat [38], is divided into three domains: core promoter (core p), upstream control region (UCE) and sequences upstream of UCE. Black dots represent methylated CpG nucleotides. Each row of dots represents one bisulfite-sequenced clone. The clones originate from two independent experiments. HEK293 (input), bisulfite sequencing of the rRNA promoter region of total HEK293 DNA. Dicer (D349), DNA immunoprecipitated with anti-Dicer antibody D349. H3K9me2, DNA associated with histone H3 dimethylated on lysine 9 (marker for inactive genes). H4ac, DNA associated with acetylated histone H4 (marker for active genes).

To find out whether Dicer is preferentially associated with active or inactive rRNA genes, we used bisulfite sequencing to analyze promoter methylation in rDNA co-precipitated with D349 antibody from HEK293 cells. For comparison, we bisulfite-sequenced rDNA immunoprecipitated with antibodies against pan-acetylated histone H4 (H4ac, a marker for active genes) and dimethylated histone H3K9 (H3K9me2, a marker for inactive genes). As expected, the anti-H4ac antibody immunoprecipitated hypomethylated rDNA while the anti-H3K9me2 antibody immunoprecipitated hypermethylated rDNA. Interestingly, D349 antibody enriched both hypo- and hypermethylated rDNA, suggesting that Dicer association with chromatin does not discriminate between active (hypomethylated) and silenced (hypermethylated) rRNA genes (Fig. 4). Although we can not rule out that hypomethylated DNA associated with Dicer represents silenced genes, this possibility is unlikely since no similar fraction of hypomethylated DNA was found in DNA associated with H3K9me2.

### No apparent involvement of Dicer in pre-rRNA processing

**Figure 5:**
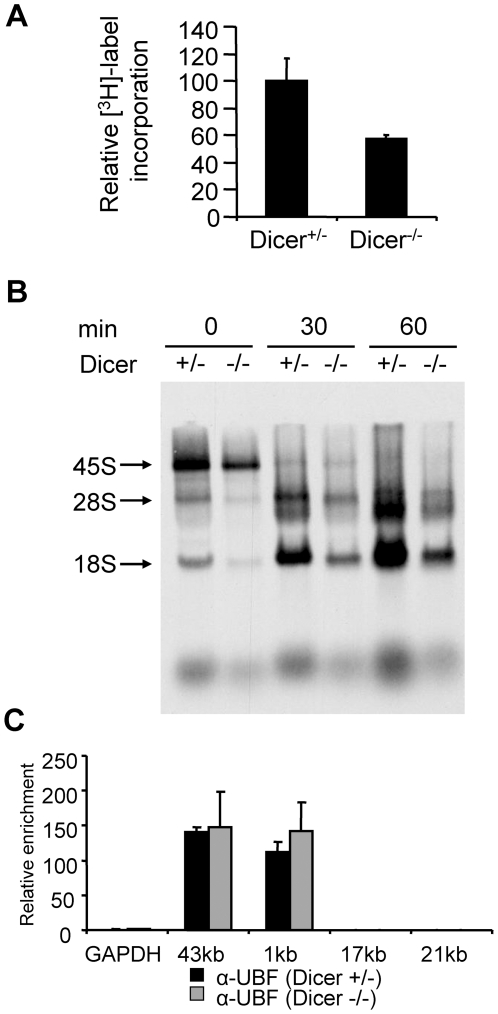
Analysis of pre-rRNA transcription and processing in mouse Dicer^+/−^ and Dicer^−/−^ ES cells. (A) Incorporation of ^3^H-methyl-label into total cell RNA. Mouse Dicer^+/−^ and Dicer^−/−^ ES cells were cultured for 30 min in the presence of [^3^H-methyl]-methionine. Incorporation measured for Dicer^+/−^ ES cells was set as 100%. The values represent means (+/− SD) from samples collected at 0, 30 and 60 min of after labeling with [^3^H-methyl]-methionine. (B) Analysis of pre-rRNA processing by agarose gel electrophoresis. Equal amounts of RNA isolated from cells cultured for 30 min in the presence of [^3^H-methyl]-methionine and chased with unlabeled methionine for 0, 30 and 60 min were separated on agarose gel and RNA was visualized by fluorography. (C) UBF association with rRNA promoter is not affected in Dicer^−/−^ ES cells. The ChIP was performed with UBF antibody [49] as described in Figure 2E. Results are shown relative to the 1∶100 dilution of respective input DNAs and represent means (+/− SEM) of at least 3 independent experiments.

To test whether Dicer plays a role in pre-rRNA maturation we analyzed pre-rRNA processing in ES cells by pulse labeling the RNA with [^3^H-methyl]methionine for 30 min and chasing the label with nonradioactive methionine for 30 and 60 min. Incorporation of the label into pre-rRNA in Dicer^−/−^ cells was about two times lower than in control Dicer^+/−^ cells (Fig. 5A). Consistently, transcriptome analysis indicated an approximate two-times lower level of 45S pre-rRNA in Dicer^−/−^ as compared to Dicer^+/−^ cells ([12], GEO Dataset GSE7141). However, as revealed by pulse chase analysis (Fig. 5B), processing of 45S pre-rRNA to mature rRNA species occurred without any obvious differences between Dicer^−/−^ and control ES cells.

### Common epigenetic marks are not affected by the loss of Dicer in ES cells

To test if Dicer localization to rDNA is associated with changes in chromatin structure and epigenetic regulations at the locus, we analyzed histone modifications and DNA methylation at rDNA in murine Dicer^−+/−^ and Dicer^−/−^ ES cells. We assessed chromatin features important for proper transcription of rRNA, such as loading of UBF [16], dimethylation of H3K4 (H3K4me2) [27] or acetylation of H4K16 (H4K16ac) [28]. We also analyzed H3K9ac, H3K9me2 and H3K9me3, modifications that have been shown to be affected by loss of Dicer in *S. pombe* [13], [29], [30].

**Figure 6:**
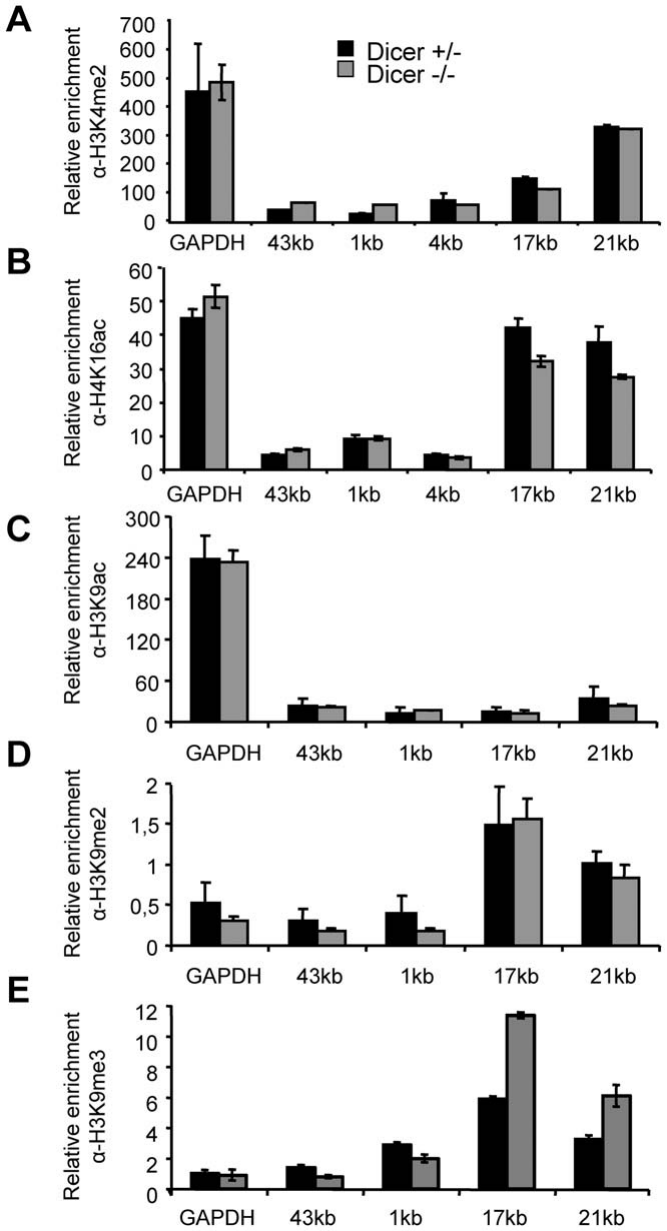
Analysis of H3K4, H4K16 and H3K9 modifications at rDNA locus. The ChIP was performed with mouse Dicer^+/−^ and Dicer^−/−^ ES cells similarly as in Figure 2, using antibodies against (A) dimethylated H3K4, (B) acetylated H4K16, (C) acetylated H3K9, (D) dimethylated H3K9, and (E) trimethylated H3K9. Results are shown relative to the 1∶100 dilution of respective input DNAs and represent means (+/− SEM) of at least 3 independent experiments.

**Figure 7:**
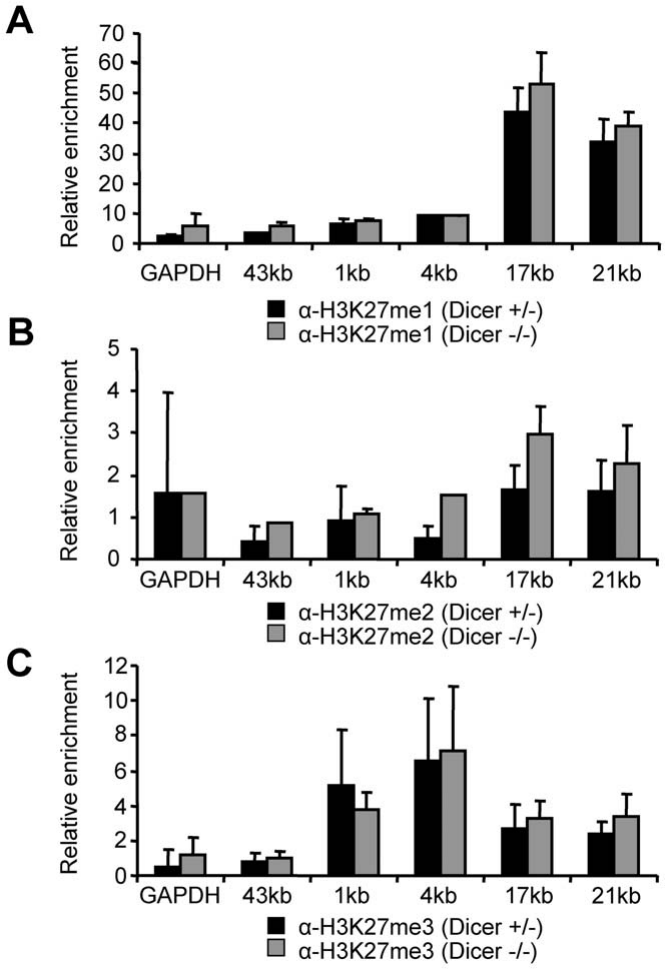
Analysis of enrichment of H3K27 methylation at rDNA locus. (A) Monomethylated H3K27, (B) dimethylated H3K27 and (C) trimethylated H3K27. Results are shown relative to the 1∶100 dilution of respective input DNAs and represent means (+/− SEM) of at least 3 independent experiments.

ChIP analysis showed that association of UBF was limited to the promoter and transcription start regions and there was no difference in the loading of UBF at the rDNA promoter between Dicer^+/−^ and Dicer^−/−^ ES cell lines (Fig. 5C). Likewise, generally, no marked differences in histone modifications H3K4me2, H4K16ac or of H3K9 were observed between Dicer^+/−^ and Dicer^−/−^ ES cells (Fig. 6A–E); an approximately 2-fold higher enrichment for H3K9me3 in the intergenic region in Dicer^−/−^ cells when compared to control cells (Fig. 6E) could be caused by Dicer-independent differences between these ES-cell lines, or could be a consequence of Dicer depletion, which is known to result in many changes in gene expression [12]. As expected for ES cells, all silencing histone methylation marks were detected at rather low levels throughout the rDNA locus [31]. In *Drosophila*, Dicer and Argonaute proteins were shown to co-localize with nuclear Polycomb protein bodies that are enriched in histone H3 methylated on lysine 27 (H3K27me) [32]. However, no pronounced differences in the mono-, di-, and tri-methylation of H3K27 were found between Dicer^−/−^ and Dicer-expressing ES cells (Fig. 7).

**Figure 8:**
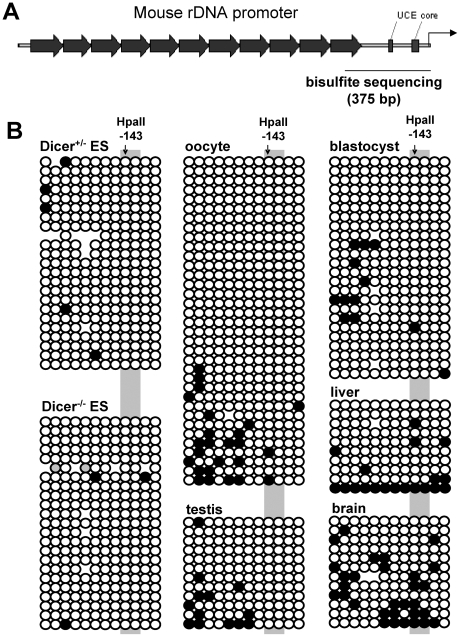
Methylation status of rDNA in mouse ES cells and selected tissues. (A) Schematic representation of mouse rRNA promoter showing position of bisulfite-sequenced region. Black arrows represent enhancer repeats, black rectangles indicate position of the UCE and core promoter elements. (B) Methylation status of rRNA promoter in mouse Dicer^+/−^ and Dicer^−/−^ ES cells, oocytes, blastocysts, liver, testis, and brain. Black dots represent methylated CpG nucleotides. Each row of dots represents one bisulfite-sequenced clone. Oocyte data were pooled from 2 independent amplifications. The redundancy of the bisulfite-sequenced clones is very low as evident from low, if any, similarity of the methylation pattern between clones containing at least one methylated cytosine.

Finally, analysis of rDNA from Dicer^+/−^ and Dicer^−/−^ ES cells by bisulfite sequencing revealed that it was largely hypomethylated, irrespective of the Dicer expression status. This contrasts with the situation in HEK293 cells in which approximately half of rDNA genes were methylated at the promoter (Fig. 5); depletion of Dicer in HEK293 cells by RNAi [33] had no effect on the DNA methylation status of rDNA (Fig. S2). Interestingly, the rRNA promoter hypomethylation was not specific to ES cells, as it was also found in primary tissues, including blastocysts (from which ES cells are derived), oocytes, and testis, brain and liver (Fig. 8). This rather unexpected finding is discussed in more detail in the Discussion. Taken together, analysis of epigenetic marks and pulse-chase analyses make it improbable that Dicer plays a role in transcriptional regulation or processing of rRNA.

## Discussion

We have found that mammalian Dicer is physically associated with rDNA chromosomal in mammalian cells. Dicer associates with both transcribed and silenced rRNA genes and it is mostly localized to rDNA region encoding to 45S pre-rRNA. Our data argue against a possibility that the rDNA-associated Dicer has a role in establishing transcriptionally repressive or permissive chromatin, or in rRNA precursor processing. Apart from the analysis of rDNA-associated Dicer, our data provide a comprehensive view of the rDNA chromatin structure in the transcribed region and ISG in mammalian cells.

Analysis of H3K9 and H3K27 modifications confirmed results of a previous study of rDNA in ES cells, which analyzed chromatin structure at the 3′ end of the transcribed rDNA region [31]. Additionally, we characterized chromatin structure of the rRNA promoter and the IGS. We found UBF present at the rRNA promoter but not in the IGS. Interestingly, the rRNA promoter had rather low levels of “active” marks, such as H3K4me2 and H3K9ac when compared to the GAPDH promoter. Repressive histone marks, such as H3K9 methylation and acetylation levels were also low. In contrast to the previous analyses of UBF binding in human HeLa and mouse A9 cells, which suggested that UBF binds throughout rDNA repeat [34], we did not observe enrichment of UBF in the intergenic spacer in mouse ES cells; similarly, Nemeth et al. [35] found that in mouse NIH 3T3 cells the UBF factor is most abundant at the rDNA enhancer/promoter region and not enriched at the intergenic spacer. Nemeth et al. [35] also provided data about occupancy of other transcription factors and on chromatin structure, including some histone modifications, at rDNA genes in NIH 3T3 cells.

It has been repeatedly documented that DNA methylation is associated with transcriptional inactivation of rDNA (for recent review, see [17]). Our data indicate that DNA methylation is not consistently found in mammalian rDNA. In fact, except of transformed cell lines, we only rarely saw clones diagnostic of hypermethylation upon bisulfite sequencing of DNA isolated from primary tissues or cells, including liver and testis, oocytes and blastocysts, and also undifferentiated and differentiated ES cells. Only in brain DNA we observed methylation of CpG at position -143 in approximately 40% of clones. However, even in this case the distribution of methylation in sequenced clones was heterogeneous, which contrasts with the two very distinct patterns of methylation in HEK293 cells (Fig. 3). A survey of the literature and our own data point to extreme diversity of the DNA methylation patterns of rDNA promoters in somatic cells. Published bisulfite sequencing data reveal three patterns of DNA methylation at rRNA promoters: (1) very low (if any) methylation in some clones and strong hypermethylation represented by long stretches of methylated CpGs in other clones [36], [37]; (2) more mosaic distribution of methylated CpGs across individual clones [38]; (3) low, if any, methylation in mouse cells and some primary human cell types (Fig. 7). Considering very different CpG density of the mouse and human rRNA promoters, we propose that CpG methylation at rRNA promoters is an accessory, possibly non-essential mechanism, which stabilizes rDNA silencing but is of variable significance in different cells of different mammalian species. rDNA methylation seems to have a stochastic cumulative nature. It accumulates to larger extend in long-established transformed cell lines such as NIH 3T3 [35], HEK293 or HeLa [36] (and this work), in non-dividing neuronal cells [37], and aged cells [39]. This suggests that the main role of rDNA methylation is long-term stabilization of the most advantageous expression level of rRNA in specific cells.

We report for the first time association of Dicer with chromatin. This association is relatively stable and remains preserved during mitosis as evidenced by staining of metaphase chromosomes. This is similar to several non-histone proteins (for example, the transcription factor UBF [40] or the chromatin remodeling protein ATRX [41]), which remain associated with rDNA chromatin during mitosis. Immunofluorescent staining of interphase cells with anti-Dicer antibodies did not provide conclusive evidence in support of nucleolar localization of Dicer because of a high background staining of whole mount cells (data not shown). In contrast to interphase cells, the signal on mitotic chromosomes is much more concentrated while the background signal is reduced by elimination of the nucleoplasm, therefore allowing for Dicer detection. It is unlikely that the observed staining of mitotic chromosomes is due to non-specific cross-reactivity of Dicer antibodies. Using different human and mouse cells, we detected the same unique staining pattern with two different antibodies raised against distinct Dicer peptides (Fig. 1). Furthermore, similar staining was observed with four different anti-tag (anti-HA, anti-FLAG, anti-Myc, and anti-EGFP) antibodies used to detect Dicer fusions with the respective tags, which were transiently expressed in HEK293 cells (Fig. 2).

Association of Dicer with rDNA presumes that Dicer enters the nucleus. Interestingly, dsRNA-binding domain (dsRBD) was recently shown to play a role in nucleo-cytoplasmic shuttling of ADAR1 [42] and we found that dsRBD of Dicer acts as a nuclear localization signal and that Dicer shuttles between the cytoplasm and the nucleus in human cells (M. Doyle and W. Filipowicz, unpublished results). Shuttling of Dicer and the role of its dsRBD in nuclear localization was also recently reported in *S. pombe* [43].

While Dicer localization to rDNA is supported by several lines of evidence, its role there remains unclear. It is unlikely that Dicer is involved in processing of pre-rRNA or its fragments into siRNA since siRNA-like small RNAs originating from rRNA and accumulating in mammalian cells were demonstrated to be produced in a Dicer-independent way [24], [44]. However, rRNA-derived small RNAs were found bound to P19 suppressor of RNA silencing known to interact with small siRNA-like duplexes [21]. In addition, small RNAs were shown to induce transcriptional silencing and changes in chromatin structure in mammalian cells [4]–[6]. Could the rDNA associated Dicer be a part of a *cis*-acting epigenetic system regulating rDNA expression by small RNAs? Several lines of evidence argue against such a role.

Regulation of rDNA chromatin is complex and involves non-coding RNAs. A recent study by Mayer et al. [20] demonstrated that 150–300 nt-long RNA transcribed from the rDNA promoter region is required for the functional integrity of the NoRC complex, which is involved in heterochromatization and silencing of rRNA genes. However, it is unlikely that Dicer plays a role in the NoRC-mediated regulation. In contrast to NoRC, Dicer association extends over an entire rDNA transcribed region. In addition, small RNAs (<25-nt; size expected for the products of Dicer cleavage) were not found among RNAs associated with TIP-5, an RNA binding protein component of NoRC [20].

Furthermore, metabolic labeling experiments performed with Dicer^−/−^ ES cells showed no apparent defect in pre-rRNA processing in these cells. Although pulse-chase experiments revealed decrease in the level of pre-rRNA synthesis in Dicer^−/−^ ES cells, this finding is readily explained by a substantially slower growth of these cells when compared to control Dicer^+/−^ cells ([12], [26]). Furthermore, we previously found that Retinoblastoma-like 2 (Rbl2), a transcriptional repressor, is inhibited in ES cells by miRNAs of the miR-290-295 cluster and, consequently, the Rbl2 level is elevated in Dicer^−/−^ cells, which are depleted of mature miRNAs [12]. Since Rbl2 overexpression has negative effect on rRNA synthesis [45], it is likely that reduced rRNA expression in Dicer^−/−^ cells is, at least partially, a consequence of the miRNA loss.

Recently, Peng and Karpen [25] reported that the RNAi machinery is responsible for proper nucleolar architecture in *Drosophila*. Loss of Dicer-2 in *Drosophila* led to a pronounced decrease in dimethylation of H3K9 at the rDNA loci in differentiated fly tissues. Dicer-2 mutant flies also showed disrupted organization of nucleoli and decreased stability of rDNA repeats. In contrast, we did not find any decrease in H3K9 methylation in mouse ES cells lacking Dicer. However, it should be stressed that ES cells do not represent an optimal system to study H3K9 methylation of the rRNA promoter as H3K9 repressive marks outside the IGS region are low, if not absent, in rDNA in ES cells (Fig, 6E, D and [31]). Although estimation of size of nucleoli in Dicer^+/−^ and Dicer^−/−^ ES cells by light microscopy suggested a small increase in size of nucleoli in Dicer^−/−^ ES cells (our unpublished results), this observation does not provide any insight into a possible role of Dicer in rDNA chromatin as the aforementioned changes might result from disregulation of miRNA repression or other factors.

Mutations in components of the RNAi machinery, such as Rdp1, Ago1 and Dcr1, exhibit increased mitotic recombination frequency of rDNA repeats also in *S. pombe* [13]. While the frequency of wild-type colonies showing a reporter gene loss at the tandem rDNA array was 3.3×10^−3^, the recombination frequencies of the RNAi mutant cells were 5- to 10-fold higher [13]. A similar role have been proposed in *N. crassa* [14]. The lack of evidence supporting involvement of Dicer in transcriptional regulation of rDNA or in pre-rRNA maturation raises a possibility that, by analogy to the situation in *S. pombe, N. crassa* and *Drosophila*, the Dicer association with rDNA in mouse and human cells reflects a role of this protein in maintaining the integrity of rDNA repeats also in mammals. While this hypothesis needs to be tested in the future, our data represent the first step towards understanding the role of Dicer association with rDNA in mammalian cells.

## Materials and Methods

### Cell culture and transfection

Human HEK293 and HeLa cells, and mouse teratocarcinoma P19 cells were maintained in DMEM supplemented with 10% fetal calf serum. The Dicer heterozygous (line D4) and Dicer-deficient (line 27H10) ES cells (referred as Dicer^+/−^ and Dicer^−/−^, respectively) were kindly provided by G. Hannon, Cold Spring Harbor Laboratory, Cold Spring Harbor, NY [26]. They were maintained on gelatin-coated plates with DMEM supplemented with 15% fetal calf serum, Na pyruvate, β-mercaptethanol, non-essential amino acids and mouse leukemia inhibitory factor. Human lymphocytes were kindly provided by A. Wodnar-Filipowicz (University Hospital, Basel). To generate plasmid pNHA-Dicer, expressing the human Dicer protein tagged at the N-terminus with the influenza hemaglutinin (HA) tag, a full-length Dicer cDNA [46] was subcloned into pCI-N-HA expression vector [47]. HEK293 cells were transfected with 1.6 µg of pNHA-Dicer per well of the 6-well plate, using Lipofectamine 2000. The Myc- and Flag- tagged human Dicer expression plasmids were kindly provided by Michael Doyle (FMI). The EGFP-tagged human Dicer was kindly provided by Maciej Drozdz (FMI).

### Cytospin and immunostaining

Cells were treated with colchicine (0.2 µg/ml for 2 h) to block the cells in metaphase. After shake-off or mild trypsinization, mitotic cells were lysed in 75 mM KCl. Chromosomes were spread on slides in the Cytospin (2,000 rpm for 10 min). After several washing steps with 10 mM Tris-HCl, pH 8.0, containing 120 mM KCl, 20 mM NaCl, 0.5 mM EDTA, 0.1% Triton X-100, and 0.1% Tween 20, chromosomes were fixed with 2% paraformaldehyde, blocked with blocking buffer (PBS containing 5% normal goat serum and 0.2% Triton X-100), and stained with indicated antibodies. The following primary antibodies were used for staining the mitotic spreads: anti-Dicer D349 and D350 (Fig. S1; [48]), anti-HA (Roche), anti-EGFP (Roche), anti- anti-Flag, anti-Myc, anti-CENP-A (MBL), and anti-UBF (kindly provided by B. McStay, University of Dundee [49]. After incubation for 2 h (at antibody dilutions of 1∶200, 1∶50, 1∶100, 1∶100 and 1∶200, respectively), slides were treated with appropriate anti-rabbit, anti-mouse or anti-sheep antibodies conjugated to ALEXA 488 or 594 (Molecular Probes; used at 1∶500 dilution). The DeltaVision system (Olympus IX70/100x objective) was used to acquire and deconvolve the images.

### Chromatin Immunoprecipitation

Chromatin immunoprecipitation (ChIP) was performed essentially as described previously [50]. Cells were cross-linked by adding formaldehyde directly to the medium to a final concentration of 1%, at room temperature. Incubations lasted for only 8 min to avoid excessive cross-linking and were stopped by adding glycine to a final concentration of 0.15 M. Cell lysates were sonicated to generate 300- to 1,500-base-pair DNA fragments. After pre-clearing the samples with Protein A Agarose (Upstate), the immunocomplexes were formed using anti-Dicer (D349), anti-UBF [49], and anti-H3K9ac, anti-H3K9me2, anti-H3K9me3, anti-H3K4me2, anti-H4K16ac, anti-H3K27me1, anti-H3K27me2, anti-H3K27me3, and anti-H4ac antibodies (all nine from Upstate), and an antibody against *Drosphila* RNA 3′-terminal phosphate cyclase (our unpublished results) which served as an unspecific control antibody. Immunocomplexes were collected with 30 µl of Protein A Agarose (Upstate). The purified DNA was used as a template for quantitative real-time PCR with ABI Prism 7000 Sequence Detection System (Applied Biosystems) and Platinum SYBR Green qPCR SuperMix (Invitrogen) together with a 1∶100 dilution of the respective input DNA. Sequences for the specific primers are given in Table S1. Annealing of all primers was at 55°C.

### FACS analysis

HEK293 cells were stained with 5 µg/ml of Hoechst33342 in DMEM for 30 min and collected in PBS contatining 3% fetal calf serum. The stained cells were sorted according to cell cycle phase with MoFlow cell sorter (Dako Cytomation) and the G1-phase cells were collected (4.5×10^6^ cells per experiment) and used for ChIP analyses.

### Bisulfite sequencing

Bisulfite sequencing was performed as described previously [51]. 200 ng of genomic DNA or DNA immunoprecipitated with a specific antibody or DNA from 30 oocytes was used as a starting material. Apart from ES cells, mouse samples were isolated from 12-weeks old C57BL/6 animals. Sequences for the primers targeting the rDNA promoter were for human rDNA: forward primer 5′-ATGTTTTCGGTTTTCGTTTTGGA-3′, reverse primer 5′-CATCCGAAAACCCAACCTCTCC-3′; for mouse rDNA: forward primer 5′-GGTTAGTTGGAGTTTTGGATTTTTTT-3′, reverse primer 5′-AAATAAACTAAACAAACAAAACAACCTTAAAT-3′. PCR amplification conditions were as described previously [51].

### Metabolic labeling

Mouse Dicer^+/−^ and Dicer-deficient ^−/−^ ES cells were cultured in a methionine depleted DMEM for 15 min. After starvation, 50 µCi/ml of [^3^H-methyl] methionine was added and cells were cultured for additional 30 min in 37°C. The cells were collected (time point 0 min) or cultured in normal DMEM for additional 30 or 60 min before collection. RNA was extracted using Trizol Reagent according to manufaturer's protocol. Incorporation of [^3^H-methyl] methionine into RNA was analyzed by liquid scintillation. For electrophoresis, equal amounts of total RNA were separated on 1% formaldehyde-agarose gel and transferred onto a charged nylon membrane (Hybond N+, Amersham). Membrane was dried and sprayed with En3hance autoradiography-enhancing spray (Perkin Elmer), wrapped into a polyethylene foil and exposed on an X-ray film (X-OMAT, Kodak) for one week.

## Supporting Information

Figure S1 Dicer antibodies used in the study. (A) Western blot analysis of a whole HEK293 cell lysate with the affinity purified antibodies D349 and D350. D349 was used in dilution 1∶5,000 and D350 in dilution 1∶1,000. Sizes of molecular weight markers (in kDa) are indicated. The 115-kDa band detected by D349 and D350 represents Dicer degradation product since its intensity was reduced in parallel with the full-length protein upon RNAi-mediated knock-down of Dicer (data not shown). Both anti-Dicer antibodies detect unspecific bands (asterisks) of approximate 40-kDa mobility. The ∼40-kDa proteins detected by D349 are different from those detected by D350 antibody since they electrophores with different mobility on a higher percentage polyacrylamide gel (data not shown). (B) HEK293 and ES cells contain different amount of Dicer. Western blots densitometry was used to compare different Dicer protein levels in HEK293 cells and ES cell lines D4 and 27H10. Note that enzymatically non-functional truncated Dicer upon Cre-mediated Dicer deletion can be detected in Dicer−/− ES cells (27H1). The same amount of lysate (total protein) was loaded in each lane. (C) Relative enrichment of rDNA obtained with D349 antibody in different cell types correlates with the level of Dicer expression in these cells. Enrichment in a non-synchronized and FACS-sorted G1 phase HEK293 cells is also compared. Black columns show rDNA enrichment after chromatin immunoprecipitation with D349. Gapdh sequences are not enriched.
(1.83 MB TIF)

Figure S2 rDNA methylation in HEK293 cells depleted of Dicer. Dicer knockdown in the previously established HEK293 2b2 cell line stably expressing anti-Dicer short hairpin RNA [1] was induced with doxycyclin for six days. Bisulfite sequencing of genomic DNA was performed as described in Material and Methods. The sequenced region is the same as one shown in Fig. 4, spanning positions −186 to +20 of the rDNA repeat [2]. It is divided into three domains: core promoter (core p), upstream control region (UCE) and sequences upstream of UCE. Black dots represent methylated CpG nucleotides. Each row of dots represents one bisulfite-sequenced clone. Supplementary references 1. Schmitter D, Filkowski J, Sewer A, Pillai RS, Oakeley EJ, et al. (2006) Effects of Dicer and Argonaute down-regulation on mRNA levels in human HEK293 cells. Nucleic Acids Res 34: 4801-4815. 2. Ghoshal K, Majumder S, Datta J, Motiwala T, Bai S, et al. (2004) Role of human ribosomal RNA (rRNA) promoter methylation and of methyl-CpG-binding protein MBD2 in the suppression of rRNA gene expression. J Biol Chem 279: 6783-6793.
(0.75 MB TIF)

Table S1 Primers used for ChIP analysis.
(0.06 MB DOC)
