# Supplementary figures and images for: Dicer Is Associated with Ribosomal DNA Chromatin in Mammalian Cells
Source: PLoS One. 2010 Aug 13;5(8):e12175. doi: 10.1371/journal.pone.0012175 (PMC2921364; doi:10.1371/journal.pone.0012175)

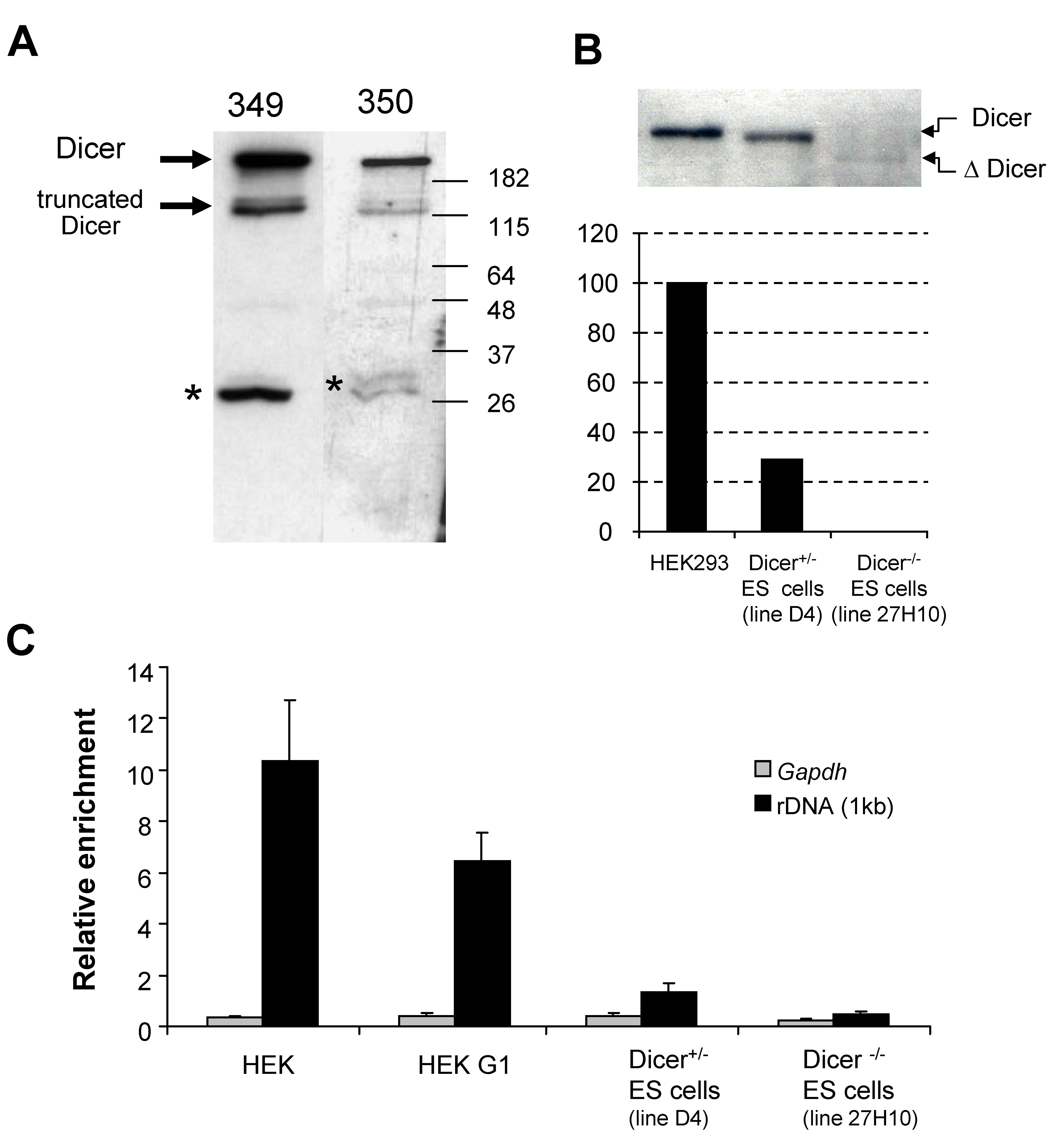

Supplement: Figure S1 — Dicer antibodies used in the study. (A) Western blot analysis of a whole HEK293 cell lysate with the affinity purified antibodies D349 and D350. D349 was used in dilution 1∶5,000 and D350 in dilution 1∶1,000. Sizes of molecular weight markers (in kDa) are indicated. The 115-kDa band detected by D349 and D350 represents Dicer degradation product since its intensity was reduced in parallel with the full-length protein upon RNAi-mediated knock-down of Dicer (data not shown). Both anti-Dicer antibodies detect unspecific bands (asterisks) of approximate 40-kDa mobility. The ∼40-kDa proteins detected by D349 are different from those detected by D350 antibody since they electrophores with different mobility on a higher percentage polyacrylamide gel (data not shown). (B) HEK293 and ES cells contain different amount of Dicer. Western blots densitometry was used to compare different Dicer protein levels in HEK293 cells and ES cell lines D4 and 27H10. Note that enzymatically non-functional truncated Dicer upon Cre-mediated Dicer deletion can be detected in Dicer−/− ES cells (27H1). The same amount of lysate (total protein) was loaded in each lane. (C) Relative enrichment of rDNA obtained with D349 antibody in different cell types correlates with the level of Dicer expression in these cells. Enrichment in a non-synchronized and FACS-sorted G1 phase HEK293 cells is also compared. Black columns show rDNA enrichment after chromatin immunoprecipitation with D349. Gapdh sequences are not enriched. (1.83 MB TIF) [file pone.0012175.s001.tif]
