# Supplementary material for: Dicer Is Associated with Ribosomal DNA Chromatin in Mammalian Cells
Source: PLoS One. 2010 Aug 13;5(8):e12175. doi: 10.1371/journal.pone.0012175 (PMC2921364; doi:10.1371/journal.pone.0012175)
Supplement: Table S1 — Primers used for ChIP analysis. (0.06 MB DOC) [file pone.0012175.s003.doc]

**Table S1. Primers used for ChIP analysis.**

| **Primers for ChIP analysis in human cells** ( For numbering of nucleotides in rDNA, see Genbank accession number U13369) | | | |
| --- | --- | --- | --- |
| Primer pair | | Product Size (bp) | Primer sequences (5´-3´) |
|  |  |  |  |
| GAPDH | forward | 153 | GAACCAGCACCGATCACCTC |
|  | reverse |  | CCGTAAAACCGCTAGTAGCCG |
| 1 kb | forward | 90 | AAAGCCTTCTCTAGCGATCTGAGAG |
|  | reverse |  | CGCTACCATAACGGAGGCA |
| 3 kb | forward | 108 | CCGCGCTCTACCTTACCTACC |
|  | reverse |  | TCGCAGTTTCACTGTACCGG |
| 6 kb | forward | 145 | CTTAGCGGTGGATCACTCGG |
|  | reverse |  | GCTCAGACAGGCGTAGCCC |
| 13 kb | forward | 161 | AAGCGTTGGATTGTTCACCC |
|  | reverse |  | CTCAGCCAAGCACATACACCA |
| 20 kb | forward | 113 | TGATGCCTTCCGTAGCCTTG |
|  | reverse |  | CCAGTTTTCAGCCCCAACAC |
| 29 kb | forward | 245 | CCACGGCTCTAGTCTGGGC |
|  | reverse |  | GGCAGCTAACGTGTCTTGGG |
| 38 kb | forward | 103 | GGTGTCCGTGCCAGTGATTC |
|  | reverse |  | CCCACCCATCGGATCATCT |
| 42 kb | forward | 83 | GTTTTGGGCACCGTTTGTG |
|  | reverse |  | GCGAAACCGTGAGTCGAGAA |
| **Primers for ChIP analysis with mouse cells** (For numbering of nucleotides in rDNA, see Genbank accession number BK000964) | | | |
| GAPDH | forward | 70 | TCCCCTCCCCCTATCAGTTC |
|  | reverse |  | TTGGACCCGCCTCATTTTT |
| 1 kb | forward | 188 | GCACGCGCTGTTTCTTGTAA |
|  | reverse |  | CCTCCTTCTCTCCTCGACCC |
| 4 kb | forward | 149 | GTAGTCGCCGTGCCTACCAT |
|  | reverse |  | TTTTCGTCACTACCTCCCCG |
| 17 kb | forward | 132 | ACCCCTTCAGAGTTTACCGGA |
|  | reverse |  | GGACCCAGGTTGGTGAGAGA |
| 21 kb | forward | 71 | AGCAAAATCCAATCCAAACTTCA |
|  | reverse |  | TGTTTTCTATTAGACGCAAGCAACTG |
| 43 kb | forward | 54 | GGCCAAAGCAGACCGAGTT |
|  | reverse |  | CCAACCCTAGCATTTTTCCCA |
